# IFN-*λ*: A New Class of Interferon with Distinct Functions-Implications for Hepatitis C Virus Research

**DOI:** 10.1155/2015/796461

**Published:** 2015-05-20

**Authors:** Bing Liu, Ian McGilvray, Limin Chen

**Affiliations:** ^1^Institute of Blood Transfusion, Chinese Academy of Medical Sciences, Peking Union Medical College, Chengdu, Sichuan 610052, China; ^2^Toronto General Research Institute, University Health Network, University of Toronto, Toronto, ON, Canada M5G 1L6

## Abstract

Pegylated interferon-*α* and ribavirin (PEG-IFN/RBV) is widely used to treat chronic hepatitis C virus infection with notorious adverse reactions since the broad expression of IFN-*α* receptors on all nucleated cells. Accordingly, a Type III IFN with restricted receptors distribution is much safer as an alternative for HCV therapy. In addition, single nucleotide polymorphisms (SNPs) near the human *IFN-λ3* gene, *IL-28B*, correlate strongly with the ability to achieve a sustained virological response (SVR) to therapy with pegylated IFN-*α* plus ribavirin in patients infected with chronic hepatitis C. Furthermore, we also discuss the most recent findings: *IFN-λ4* predicts treatment outcomes of HCV infection. In consideration of the apparent limitations of current HCV therapy, especially high failure rate and universal side effects, prediction of treatment outcomes prior to the initiation of treatment and developing new alternative drugs are two important goals in HCV research.

## 1. Introduction

**Figure 1:**
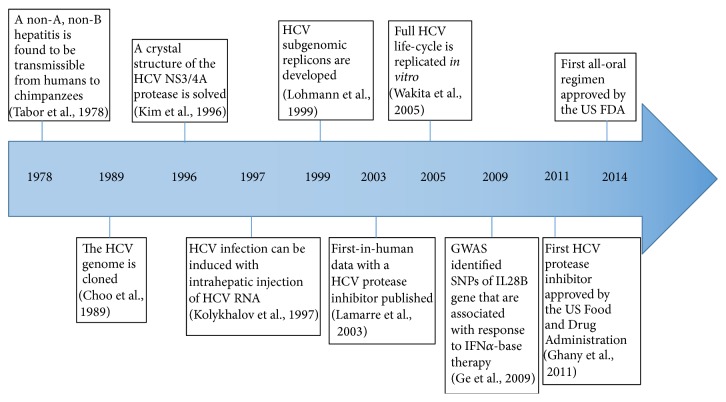
Timeline | Key discoveries in the basic science of HCV.

More than 30 years ago, a blood-borne non-A, non-B hepatitis was discovered and the virus was subsequently named as hepatitis C virus (HCV) (Figure 1). Currently, an estimation of 130–170 million people are chronically infected with HCV worldwide, which is a growing global pandemic and financial burden to the society [1]. Current standard of care (SOC) is the combination therapy with pegylated IFN-*α* and ribavirin (PEG-IFN/RBV) in developing countries, while DAAs in combination with PEG-IFN/RBV (triple therapy) significantly improve the SVR to some extent in developed districts [2]. Nevertheless, in consideration of morbid side effects, variable cure rates, and high costs, it is very important to predict treatment response and identify critical insights into mechanism of viral resistance. In this review, we will discuss biology and signaling pathway of the IFN-*λ*s, as well as their utility in clinic trial of anti-HCV therapy and link with treatment-induced clearance of HCV for prediction of treatment outcome.

## 2. A Brief History of IFN-*λ*

### 2.1. Classification

In 2003, the first 3 members of the IFN-*λ* family (IFN-*λ*1, IFN-*λ*2, and IFN-*λ*3) were uncovered by 2 independent groups based on genomic sequence [3, 4]. With the development of genome-wide association studies (GWAS), Prokunina-Olsson and his colleagues discovered a novel IFN-*λ* gene,* IFN-λ4*, which is located in between* IFN-λ2* and* IFN-λ3* [5]. IFN-*λ*4 is a low-level-expression protein in a small fraction of the human population which is different from other IFNs. Its expression depends on polymorphism* ss469415590*, which is in linkage disequilibrium (LD) with* rs12979860*. IFN-*λ*4 protein can only be produced by individuals who carry the* ΔG* allele of the* ss469415590* variant (*IFN-λ4-ΔG*), while the major* TT* allele disrupts the IFN-*λ*4 ORF due to a frame shift [6]. While being structurally related to IL-10-related cytokines, IFN-*λ*s have been functionally classified as a distinct type of IFN because they signal through binding to IFN*λ* receptor to exert the antiviral activity [7].

### 2.2. IFN-*λ* Receptor Distribution

IFN-*λ*s bind and signal through a heterodimer receptor composed of a short IL-10R2 chain (also called IL-10Ra) and a long chain IL-28R1 (also called IL-28Ra) [3, 4]. Although the short chain is ubiquitously expressed and is a primary part of the receptor complexes for IL-10, IL-22, and IL-26, the long chain is utilized only by IFN*λ* and has a limited tissue distribution [8]. Broadly speaking, Type III IFNs are similar to Type I IFNs (IFN-*α*/IFN-*β*) in that they both have antiviral and immunomodulatory properties. On the other hand, they are quite different. While Type I IFNs (IFN-*α*/IFN-*β*) are ubiquitously expressed by most somatic cells, Type III IFNs can only be expressed by some cell types, such as epidermal, bronchial, and gastrointestinal epithelial cells [9]. In addition, IFN-*λ* receptors are not found on fibroblasts [10], microvascular endothelial cells, adipocytes [8], or primary CNS cells [11]. IL-28R1 was shown to be expressed on the surface of some circulating immune cells including B cells, T cells, DCs [12, 13], and macrophages [14]. Nevertheless, these cells expressed a short IFN-*λ* receptor splice variant (sIFN-*λ*R1/sIL-28R1) to reveal a secreted, glycosylated protein to inhibit IFN-*λ* signaling through binding IFN-*λ*1 with a moderate affinity (*K*_*D*_ 73 nM) [8]. Therefore, there is no consensus conclusion about receptor levels on the surface of immune cells, which are an influential target of IFNs in general [15].

### 2.3. IFN-*λ*-Mediated Signaling Pathway

**Figure 2:**
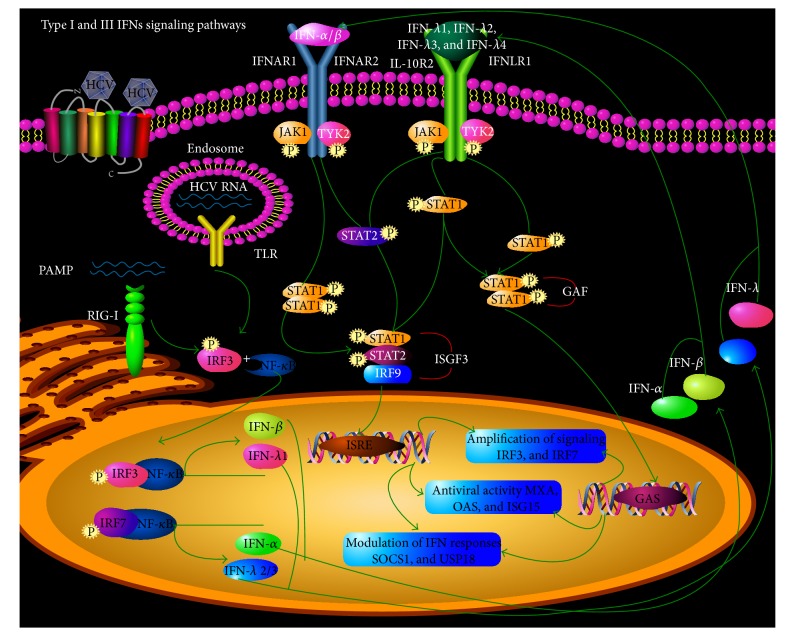
Types I and III IFNs canonical signaling pathways. Viruses, including HCV, are recognized by pattern recognition receptor (PRR), TLR3, and/or RIG-I-like receptor, leading to the activation of kinases. This in turn results in phosphorylation of IRF3 and IRF7 and activation of NF-*κ*B. They translocate to nucleus to form heterodimers, respectively, which can catalyze transcription of IFN-*α*, IFN-*β*, and IFN-*λ* genes by binding to specific DNA sequences. Then, Types I and III IFNs move out of nucleus to bind to their specific receptors on the cell membrane and trigger an overlap pathway, Jak/STAT signaling pathway. Upon binding to their cognate receptors, Type I can phosphorylate both STAT1 and STAT2 to form ISGF3 that binds to ISRE in the promoter region of ISGs to upregulate their transcription and Type III IFN also can phosphorylate STAT1 to form a homodimer GAF and induce ISGs expression with GAS in the promoter region. A myriad of ISG products is not only antiviral factors but also participation in the signaling pathway in virtue of positive/negative feedback.

Although they signal through distinct receptors, Type I and Type III IFNs trigger remarkably similar responses in cells through activation of the Jak/STAT pathway (Figure 2). Viral nucleic acids are sensed by transmembrane Toll-like receptors (TLRs), cytoplasmic DNA sensors, and RNA helicases, resulting in the activation of kinases to initiate signaling cascade [16]. The kinases activate NF-*κ*B, IRF3 and IRF7 transcription factors to induce their subsequent translocation into the nucleus where they promote IFN gene transcription [17]. The complex of IRF3 and NF-*κ*B stimulates expression of IFN-*β* and IFN-*λ*1, while the complex of IRF7 and NF-*κ*B stimulates the expression of IFN-*α* and IFN-*λ*2 and IFN-*λ*3 [18]. Once produced, Types I and III IFNs translocate from the nucleus to the cytomembrane to bind their receptors, respectively, to activate the overlapping Jak/STAT signaling pathway. Type I IFNs use a dimeric receptor (IFNAR) composed of subunits IFNAR1 and IFNAR2c, whereas Type III IFNs signal through a different receptor, which is composed of IL-28Ra and IL10Ra. After IFNs bind to their specific receptors, the receptor-associated tyrosine kinases, JAK1, TYK2, are activated to further simulate phosphorylation of STAT proteins. On the one hand, activated STAT1, STAT2, and IRF-9 form a heterotrimeric transcription factor complex called IFN-stimulated gene factor 3 (ISGF3). ISGF3 is then translocated into the nucleus where it binds to sequences of IFN-stimulated response elements (ISRE) present in the promoter region of numerous interferon stimulated genes (ISGs) [19]. On the other hand, a homodimer STAT1 (gamma interferon activation factor, GAF) is formed and translocated into the nucleus where it binds to GAS (gamma interferon activation site) to induce ISGs expression [20, 21]. The proteins encoded by these ISGs have different biological functions. Some mediate a myriad of antiviral activities, such as ISG15, MxA, and OAS. Several ISG-encode proteins like IRF7, IRF3 amplify IFNs production through a positive feedback [22]. Nevertheless, ISG-encoded proteins like USP18 and SOCS1 act as negative regulators for IFN signal pathway [23, 24].

## 3. Clinical Applications of IFNs for HCV Treatment

### 3.1. Current Treatment Regime for HCV Infection

With the development of recombinant IFNs, IFN-*α*-based treatment has formed the cornerstone for the treatment of HCV infection for the past two decades [25]. Until now the standard of care (SOC) for HCV in most developing countries is weekly subcutaneous injection of pegylated IFN-*α* (PEG-IFN-*α*) combined with daily ribavirin [26]. However, the traditional success of IFN-*α*-based treatment has been challenged by suboptimal SVR rates in treatment resistant patients, cumbersome treatment regime, and deleterious side effects [4]. The recently successful developments of numerous well-tolerated oral agents that target viral proteins to interfere with viral replication and direct antiviral agents (DAAs) increase SVR in patients infected with HCV [27]. There are four major groups of DAAs: NS3/4A protease inhibitor (PIs), NS5B nucleoside polymerase inhibitors (NPIs), NS5B nonnucleoside polymerase inhibitors (NNPIs), and NS5A inhibitors [28]. In 2011, the first DAAs telaprevir and boceprevir were approved by FDA. Combined with PEG-IFN/ribavirin treatment, these DAAs significantly increased the SVR up to 80% for the most prevalent HCV genotype 1 infected patients although they had marginal improvement of SVR in other genotypes [29, 30]. Currently, several promising DAAs are already in clinical trials as a monotherapy to translate the dream of a pill to cure HCV into reality (reviewed in [31]). FDA approved first the monopill, Harvoni (ledipasvir and sofosbuvir), without combination of interferon or ribavirin to treat HCV genotype 1 infection in October 2014. Indeed, IFN-free regimen is beneficial for patients with poor tolerance to IFN and/or who have suffered from extensive side effects. Nevertheless, the apparent limitations of DAAs include low genetic barrier to resistance and high cost. As such, interferon will still be used to treat HCV infection in the immediate future, especially in most developing countries.

### 3.2. Potential Clinical Application of IFN*λ* for HCV

Despite the availability of new DAAs, IFN-a-based treatment remains an effective therapy for HCV infection in most developing countries. However, in consideration of increasing viral resistance and undesirable systemic side effects due to the fact that virtually all cell types express IFNAR [32], other alternatives with better efficacy and less toxicity should be explored. As a consequence, IFN-*λ*s, which trigger the overlapping Jak/STAT signaling pathway with IFN*α* through distinct IFN-*λ*R1 expressed only in restricted tissues [33], seem an ideal therapeutic candidate for HCV therapy. A more limited target cell range would be of great importance for the possible regimen applications of IFN-*λ*s. Refractory state occurs commonly in cultured cells and in the liver through repeated stimulation with IFN-*α* [34], which is believed to be one of the reasons for nonresponse to the treatment with IFN-*α*-based therapy in HCV-infected patients [35]. Surprisingly, IFN-*λ* did not induce such a refractory state in liver cells [36]. That might be because tolerance of IFN-*α*-based therapy correlated with refractoriness of NK cells [37] where no expression of IFN-*λ*R1 was detected [38]. Although selective distribution of the IFN-*λ* receptors would reduce the number of potential medical indications, fewer side effects would represent a big advantage over type I IFNs [39]. Preclinical studies showed that weekly PEG-IFN-*λ* with or without daily RBV inhibited HCV replication in human hepatocytes with minimal adverse events and hematologic effects in patients with chronic HCV [40]. Furthermore, Muir and his group demonstrated that antiviral effects of recombinant IFN-*λ*1 is comparable to recombinant IFN-*α* in clinical trial [41]. In recent phase 3 clinical trials, IFN-*λ*1a was recommended as 180 mg doses in combination with ribavirin and a direct-acting antiviral for 24–48 weeks in HCV genotype 1 or genotype 4 or 12–24 weeks in genotype 2 or genotype 3 [42, 43]. Alternative treatment like IFN-*λ*s that target the host immune response with fewer side effects due to limited receptor distribution becomes a promising strategy.

## 4. Prediction of Treatment Response in Patients Infected with Hepatitis C Virus

Although some viral and host factors that are associated with viral clearance were identified, predication of response to therapy was suboptimal. It has been shown that host gene polymorphism plays an essential role in determining treatment outcomes of IFN-*α*-based therapy. Ge and colleagues in 2009 discovered that single nucleotide polymorphisms (SNPs) linked to the* IFN-λ3* (also known as* IL28B*) was associated with the spontaneous and treatment-induced clearance of HCV infection [44]. Interestingly, patients with upregulation of ISGs in liver cells prior to receiving interferon treatment respond less well to interferon and are much more likely to have the unfavorable* IL-28B* genotype [45, 46]. Furthermore, the polymorphism of new gene* IFN-λ4* is strongly associated with impaired spontaneous HCV clearance, which serves as an important predictive biomarker for treatment outcomes [47, 48]. Therefore, it is not surprising that IFN-*λ* opened a new era for HCV therapy as well as for the prediction of treatment outcomes.

## 5. Viral Factors to Predict IFN-based Treatment Response

### 5.1. HCV Genotype

HCV contains six major genotypes (1–6) that differ from each other by 30–35% of nucleotide sequence [49]. Among the factors identified to influence treatment outcomes, viral genotype was recognized to be of major importance. Numerous studies indicated that the SVR of 80% for those infected with genotype 2 or genotype 3 are achieved, but 40–50% for patients infected with HCV genotype 1 or genotype 4 [50–52]. Thus, HCV-1 and HCV-4 are defined as difficult to treat genotypes. While with recent high-speed development of DAAs, the SVR of genotype 1 has been significantly increased [53]. Therefore, genotype 4 was the final piece of the puzzle to solve the problem. Although the key to the puzzle is unclear, it is indicated that dynamics of HCV genotype 4 are rather tardive, which are equal to those of HCV genotype 1 and slower than those of HCV genotype 2 [54]. In conclusion, genotype is one of the most significant viral factors in determining treatment outcomes of IFN-*α*-based therapy.

### 5.2. Viral Load and Gene Mutations

This is paradoxical since a higher pretreatment viral load has been associated with a poor response to subsequent IFN-*α* treatment. Besides, it is well-known that the viral load reflects the intricate virus-host interaction which can be affected by HCV genotypes. Thus, the pretreatment baseline and on-treatment viral load can be utilized to predict SVR. Currently, a rapid virological response (RVR) at week 4 of therapy has been identified as an important predictor of SVR in patients infected with HCV genotype 1 [55] or genotype 2 [56]. Meanwhile, more studies are looking for much earlier viral response after initiation of therapy to predict SVR [57], and even a prediction model was used [58].

In addition, HCV gene mutations also contribute to treatment response, of which the hot spot of mutation was focused on viral nonstructural protein 5A (NS5A). At least 3 functional domains of NS5A were involved in IFN resistance: ISDR (Interferon Sensitivity-Determining Region), PKRBD (PKR Binding Domain), and V3 (Variable region 3) at the C terminus [59]. Enomoto et al. further demonstrated that 4 or more mutations in the NS5A region (known as “mutant type”) were associated with high SVR rate in Japanese patients chronically infected with HCV genotype 1b [60]. There is still a subject of long controversy among different research groups [61, 62]. Although the core region of HCV is conserved, mutations of amino acid (aa) 70 and aa 91 affected SVR rate [63, 64]. However, the predictive effect of mutations in the HCV core region was only observed in HCV genotype 1b and this prediction ability still remained elusive in other genotypes and subtypes.

## 6. Host Factors to Predict IFN-Based Treatment Response

### 6.1. Pretreatment ISG Expression Levels in the Liver and Blood

IFN*α* and IFN*λ* ultimately play their roles through the upregulation of ISGs. It has been shown that cultured cells enter an IFN-desensitized state that can last up to several days shortly after IFN exposure because ISGs expression is already maximally stimulated and is therefore unresponsive to further exogenous IFN-*α* treatment [65]. Therefore, the expression level of ISGs in pretreatment liver or blood seems to be useful for prediction of treatment outcomes in patients with HCV. Indeed, it has been repeatedly demonstrated that a poor response to exogenous IFN-*α* treatment is associated with a higher intrahepatic ISGs expression before treatment [66–68]. Furthermore, with the development of microarray gene-expression profiling, a high throughput method that allows simultaneously examine gene expression at the transcript (mRNA) level, it is much easier to assess the host response to HCV infection at the whole genomic scale [68]. By comparing the pretreatment hepatic gene expression levels between treatment responders and nonresponders of patients chronically infected with HCV, Chen et al. [46, 69] identified 18 genes (out of 19,000 host genes or transcripts), whose differential expression levels are associated with treatment outcomes. More interestingly, they further demonstrated that the cell-type specific expression of ISGs was correlated well with treatment outcomes, with prediction accuracy higher than that predicted by the polymorphism of IL-28B [70, 71].

Although correlation between increased pretreatment ISGs expression in liver and failure of anti-HCV therapy was identified, it is necessary to develop an easier noninvasive predictive test. In addition to the liver tissue, numerous researches revealed that blood samples might be a perfect alternative [72, 73]. Rallon et al. demonstrated that assessment of HCV/HIV coinfected patients with PEG-IFN/RBV therapy in that PBMC specimens can reliably be used for evaluating ISGs expression in clinical regardless of IL-28B genotypes [74]. In addition, wide application of DAAs urges us to develop a predictive biomarker to predict treatment outcomes in order to save expensive cost of DAAs therapy. Meissner et al. [75] conducted a clinical trial using the DAA sofosbuvir plus ribavirin (SOF/RBV) and performed detailed mRNA expression analysis in liver and peripheral blood from 60 patients who achieved either a sustained virologic response (SVR) or relapsed. They found that viral clearance was associated with rapid downregulation of IFN-stimulated genes (ISGs) in liver and blood, whereas the exact association between the expression level of ISGs in pretreatment and response to DAAs therapy would not be identified without more clinic trails.

### 6.2. Host Interleukin-28B (*IL-28B*) Genotype and SVR

Over the last decade, a myriad of host factors have been shown to play an important role in predicting the clinical outcomes of HCV by virtue of in-depth understanding of human genome and technology progress such as microarray and genome-wide association studies (GWAS). These studies accessed common SNPs among the host genome by means of a disease library, such as patients and healthy volunteers, without hypothesis based on background knowledge. Therefore, they independently uncovered the influence of* IL-28B* SNP on treatment-induced and spontaneous HCV clearance. The first landmark research facilitated by GWAS was published in 2009 [44]. Ge's group assessed the treatment outcome in a group of 1671 HCV genotype 1 patients with treatment of injecting PEG-IFN/RBV. There was a significant association between SVR and* IL-28B*,* rs12979860* SNP. Patients with allele (*C*/*C*) had rather higher SVR rate (78%) than those with allele (*T*/*T*) (28%) as well as heterozygote gene (*T*/*C*) (38%). In addition, they further compared the efficacy of PEG-IFN/RBV treatment in association with* IL-28B* genotype in different ethnicity. They concluded that more favorable IL-28B genotype was found in European than African populations, which explain higher SVR in European-Americans to some extent. However, compared with factors associated with viral clearance, host* IFN-λ3* genotype was more important than baseline viral load, the degree of liver fibrosis, or ethnicity. In subsequent years, different researches drew consistent conclusion that SNPs of* IL-28B* is strongly associated with HCV treatment outcomes [76, 77]. In addition, the verdict was extended to both HCV monoinfected and HCV/HIV coinfected populations. They revealed that the minor allele of* Rs8099917* was identified in 58% of patients who did not respond to treatment and defined as a risk factor related to progression to chronic HCV infection, regardless of the coinfection with HIV or not [78].

### 6.3. *IFN-λ4* Genetic Variation and Response to Treatment

Recently, a new variant of* IFN-λ4*, denoted as* ss469415590* (*TT*/*ΔG*) has been discovered, whose* ΔG* allele is strongly associated with impaired spontaneous HCV clearance as a result of high expression of IL-28B [47]. Interestingly,* ΔG* allele of* ss469415590* expressed a novel IFN-*λ*4, which could block the activity of Types I and III IFNs, decreasing the capacity of HCV clearance [79]. However, the mechanism of its negative regulation of IFNs signaling pathway was elusive. Considering poorly expression and antiviral activity of IFN-*λ*4, impeding receptor binding of other members of the IFN-*λ* family is a possible explanation [80]. In any event,* ss469415590* sounds like a better predictor of SVR than the traditional* rs12979860*. However, the predictive effect of* ss469415590* is still controversial (Table 1). Therefore, although the sensitivity, specificity, and predictive value of them currently identified are too low to be clinically useful alone, these studies confirmed a significant association between* IFN-λ4* genetic variation and response to treatment. Further studies should be done to explore the mechanism underlying this close association.

## 7. Prospective

Currently, there is no vaccine for HCV infection and the SOC is PEG-IFN/RBV in most developing countries. With the ever-increasing number of DAAs in development and the amazing rate of SVR reported, the era of interferon as a hallmark therapy for HCV is apparently nearing its end in developed districts regardless of the astounding cost. In contrast, despite morbid side effects and variable cure rates, IFN-*α* based treatment is still a dominant therapy in most developing countries due to cost containment. In addition, it is necessary to understand molecular mechanisms of virus-host interactions and to predict treatment outcomes before initiating therapy. Further studies need to be focused on an overall pattern taking both viral and host factors into account, which may be more reliable to predict HCV treatment response rather than one or two independent predictors. As a consequence, it is not surprising that IFN-*λ*s opened a new era as an ideal alternative for IFN-*α* with less side effects and viral resistance as well as for prediction of treatment outcomes.

## Figures and Tables

**Table 1:** Genetic association studies of *IFN-λ4* and treatment outcomes.

Single nucleotide	Subjects	Conclusions	Reference
*ss469415590* and *rs12979860 *	169 African-American patients HCV-1	Compared to *rs12979860*, *ss469415590* is more strongly associated with HCV clearance in individuals of African ancestry rather than in Europeans and Asians	[5]

*ss469415590* and *rs12979860 *	272 Caucasian patients HCV-1/HCV-4	The *ss469415590* variant shows an equivalent performance to predict SVR to pegIFN/RBV with *rs12979860 *	[81]

*ss469415590 *	80 HCV patients and 78 liver donors	lower expression of IFN-*λ*4 mRNA was associated with a higher rate of SVR in response to pegIFN-*α* and ribavirin therapy	[82]

*rs12979860 *	362 HCV-1 patients in a phase 2b trial of faldaprevir and deleobuvir	SVR in response to faldaprevir and deleobuvir was lower in the patients with the CT or TT genotypes for *IFN-λ4 rs12979860 *	[83]

*ss469415590* and *rs12979860 *	207 HCV/HIV-1 coinfected patients treated with PEG-IFN/RBV therapy	*ss469415590* genotype was a better predictor of treatment failure than *rs12979860 *	[84]

*ss469415590*, *rs8099917*, *rs12979860* and *rs12980275 *	280 HCV patients treated with PEG-IFN/RBV	*ss469415590* is superior to other *IL-28B* variants especially in patients with advanced fibrosis	[85]

*ss469415590* and *rs12979860 *	225 Thai HCV-1/HCV-3/HCV-6 patients treated with PEG-IFN/RBV	*IFN-λ3 (IL28B)* and *IFN-λ4* polymorphisms are associated with treatment response in Thai patients infected with HCV genotype 1, but not with genotypes 3 and 6	[86]

*rs12979860 *	115 HCV-1 patients treated with sofosbuvir	The *IFN-λ4-ΔG* allele was associated with slower early viral decay	[75]
